# Simulation of strawberry yield using dry matter distribution based on the potential growth of the sink–source organs

**DOI:** 10.3389/fpls.2025.1544735

**Published:** 2025-07-22

**Authors:** Tomomi Sugiyama, Yusuke Kakei, Yasunaga Iwasaki, Atsushi Oda, Masahide Isozaki

**Affiliations:** ^1^ Institute of Vegetable and Floriculture Science, National Agriculture and Food Research Organization, Tsukuba, Japan; ^2^ School of Agriculture, Meiji University, Kawasaki, Japan

**Keywords:** *Fragaria × ananassa*, greenhouse, crop model, validation, yield prediction

## Abstract

Strawberry, a vital crop in horticulture, faces challenges like pest infestations and climate variability that affect stable production. A crop model based on photosynthesis-derived dry matter (DM) production is an effective method to examine the environment–plant growth relationship. The developed model simulates total DM production and yield overtime using greenhouse environment, each inflorescence anthesis dates, leaf area, and physiological parameters as inputs. Total DM production was accurately simulated by inputting leaf area measured by either destructive measurement or web-camera based imaging without destructive measurements (RRMSE = 0.15 and 0.17). Cumulative yields closely matched measured values across two distinct growing seasons (RRMSE = 0.11–0.15). The monthly yield generally aligned with the observed values, except at the beginning and end of the harvest period, where the model tended to overestimate production. These result suggested the process of DM distribution calculation based on the potential growth of the individual leaves and fruit clusters present on that day was effective in capturing the dynamics of DM distribution to the fruit. The model could be applied to strawberry production in greenhouses controlled with optimal ranges for the plant growth. The model’s applicability to diverse greenhouse conditions would be broadened by improving the physiological parameters in future work.

## 1 Introduction

Strawberry (*Fragaria* × *ananassa*) production has increased globally, reaching 9.7 million tons in 2021, reflecting significant growth and interest in recent decades (FAOSTAT, 2023). Strawberries are cultivated in temperate and subtropical regions and even in high-altitude tropical areas (Hancock, 2000). To meet rising demand, plant management techniques have been developed worldwide (Hernández-Martínez et al., 2023). Nonetheless, commercial growers face challenges from variable weather, pests, and diseases, as strawberries are high-input, high-value crops (Hopf et al., 2022a; Hodgdon et al., 2024). Strawberry plants grow from autumn to spring, experiencing large fluctuations in temperature and solar radiation. Understanding the relationship between weather conditions and plant growth or yield aids farmers in making strategic environmental management and marketing decisions (Hopf et al., 2022b; Unnikrishnan et al., 2024).

Since the 1970s, models predicting plant growth and yield based on dry matter (DM) production—a key photosynthesis product and main component of plant dry weight (DW)—have been developed for horticultural crops (Gallagher and Biscoe, 1978). Yield is influenced by the distribution of photoassimilates to fruits. DM-based models use yield components (e.g., intercepted light, leaf area, light-use efficiency) to simulate growth under different climates (Higashide and Heuvelink, 2009). This approach enables assessing the effects of greenhouse environments on plant growth (Pérez de Camacaro et al., 2002; Higashide, 2022).

Strawberry yield models have utilized meteorological data (Pathak et al., 2016), field-scale machine learning (Maskey et al., 2019), and plant growth equations (Hopf et al., 2022a). While Hopf et al. (2022a) included leaf area in a vegetative growth model that effectively simulated total yield, leaf area and aboveground DW were overestimated, likely due to inaccuracies in DM distribution, leaf photosynthesis, and specific leaf area parameters. Precise DM calculations are crucial for photosynthesis-based yield prediction. Leaf area index (LAI), a key determinant of DM, is challenging to predict due to its dependency on factors like leaf morphology; errors in LAI lead to inaccuracies in DM production and yield. Destructive and nondestructive methods exist for determining leaf area, the latter involving web camera-based imaging to measure direct light-intercepted LAI (iLAI; Saito et al., 2022). This study examined the use of LAI or iLAI as input variables for the developed model.

DM distribution vary with the presence of competing sink–source organs in the plant (de Koning, 1994). For example, in tomatoes, a stem with three leaves preceding a truss is clustered as a vegetative unit, and fruits are considered individually (Heuvelink, 1997). In contrast to tomatoes, strawberries have a more complex structure; the stem-like structure known as the crown forms the base of the plant, and leaves and inflorescences emerge from the crown (Hidaka et al., 2018). This structural complexity makes it more challenging to define the functional units that represent the source–sink relationships in the model. Furthermore, strawberry fruit is known to be a strong sink organ for photoassimilates, which accumulates 40%–50% of the total plant DW (Nishizawa, 1994). Developing a model for yield prediction requires an accurate DM partitioning approach for vegetative and generative organs.

This study aimed to develop a DM production-based model for strawberry yield prediction, focusing on accurately simulating DM distribution to aboveground sink and source organs for observed yield alignment.

## 2 Materials and methods

### 2.1 Data acquisition for modeling

**Figure 1:**
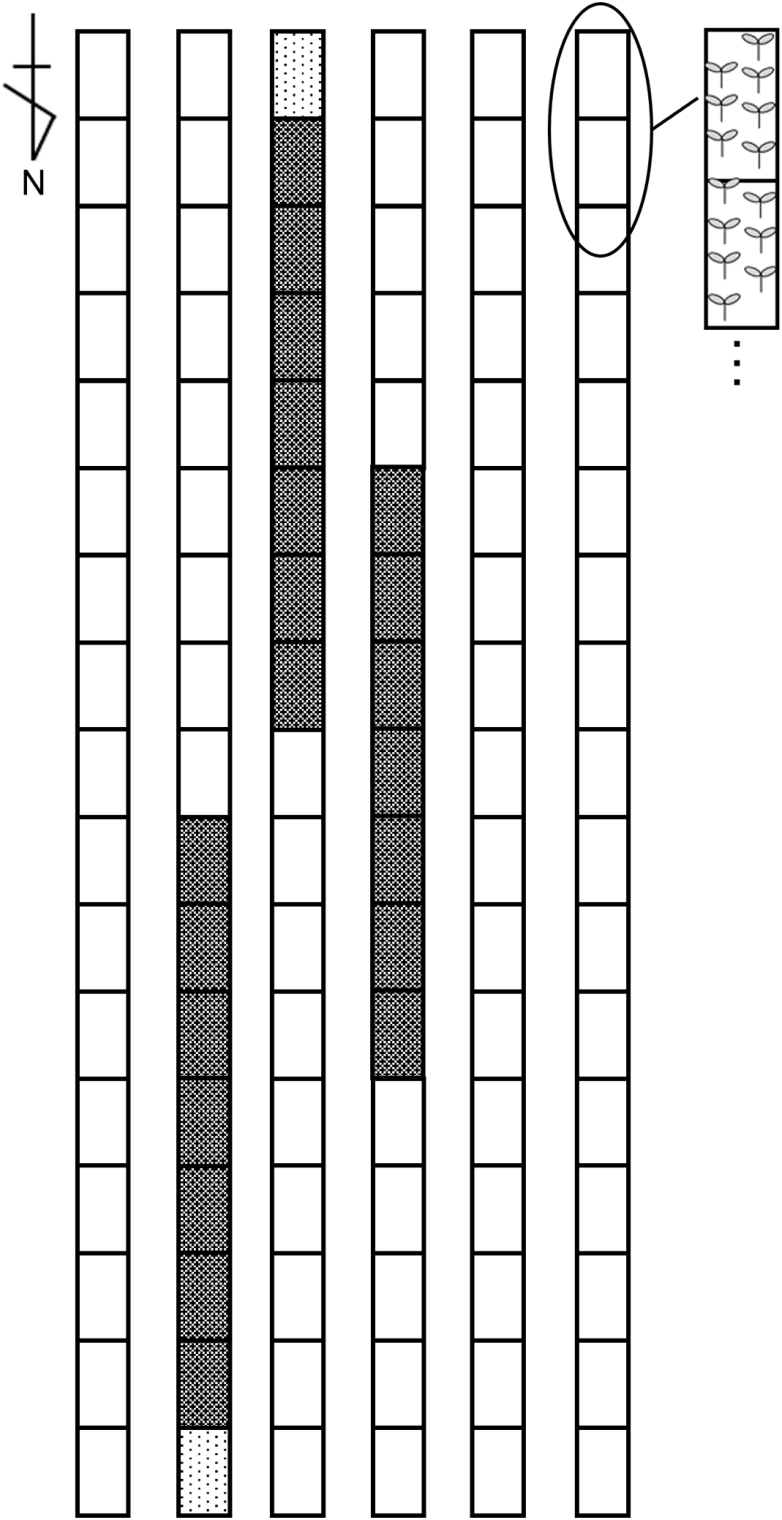
Experimental plot layout within the greenhouse. The black mesh area represents the experimental plot with ‘Benihoppe,’ the black dotted area represents the border plot of ‘Benihoppe,’ and the white area represents plots with other strawberry cultivars.

The experiment was conducted from September 18, 2019, to April 8, 2020, in a naturally ventilated greenhouse (9 × 18 × 4.5 m) at the National Agriculture and Food Research Organization, Tsukuba, Ibaraki, Japan. The June-bearing strawberry cultivar ‘Benihoppe’ (MIYOSHI AGRI-TECH CO., Ltd., Yamanashi, Japan) was used. Plants in 7.5-cm pots were maintained in the greenhouse and then transplanted to a Styrofoam planter (750 × 349 × 140 mm; Toyotane Co., Ltd., Aichi, Japan) with BVB substrate on September 18. Seven plants were arranged in double rows with 21 cm spacing, resulting in a density of 7.05 plants·m^−^². The experiment used a randomized block design with three replicates of 49 plants each, surrounded by additional strawberry plants at the same density (**Figure 1**).

An environmental control system (Priva Office, Priva, De Lier, the Netherlands) managed the greenhouse conditions: ventilation was started at 25°C during daylight hours, with windows closed at 15°C, and 80% relative humidity was maintained using a fogging system. In winter, heating activated below 6°C. Plants received a commercial nutrient solution (OAT-Agrio-A; OAT Agrio Co., Ltd.) with a pH of 5.8 and electrical conductivity (EC) of 0.65–0.90 dS·m^−^¹. Drainage EC and volume per row were monitored daily (Agrilog; ITKOBO-Z Co., Ltd., Aichi, Japan), keeping drainage EC at 0.40–0.50 dS·m^−^¹ and the daily drainage rate at 10%–30%. Beeflies (API Co., Ltd., Gifu, Japan) pollinated until November, after which honeybees were introduced. Only the main crown was retained by pruning branch crowns throughout cultivation.

Nondestructive measurements, including leaf length, flowering date, and fruit weight, tracked plant growth. Leaf length (blade and petiole) was measured twice weekly from the day when the leaf tips reached 3 cm until elongation stopped. Three plants per plot were randomly selected, measuring seven leaves on each from October to February, for a total of 21 leaves. The dates of first flower anthesis in each inflorescence were recorded for seven plants in triplicate twice or three times a week, along with yield data. All mature fruits were harvested, and the fresh weight of each inflorescence was recorded twice or three times weekly. Five marketable fruits were selected from the harvest, and dry matter content (DMC) was measured twice a month by drying at 105°C for 72 h.

Destructive measurements were taken on specific dates: planting (0 DAT), first flower anthesis (55 DAT), and first ripening in the 1st, 2nd, and 3rd fruit clusters (92, 148, and 182 DAT, respectively). Two adjacent plants per block were randomly sampled to determine the number of leaves and inflorescences, leaf area (measured with a LI-COR 3100 Leaf Area Meter; LI-COR Inc., Lincoln, NE, USA), and DW of leaves, crown, peduncle, and immature fruits. Materials were dried at 105°C for at least 72 h. Harvested fruit DW was calculated as cumulative yield to each sampling date × DMC, with total DM as the sum of aboveground parts, harvested fruits, and pruned leaves.

### 2.2 Model description

**Figure 2:**
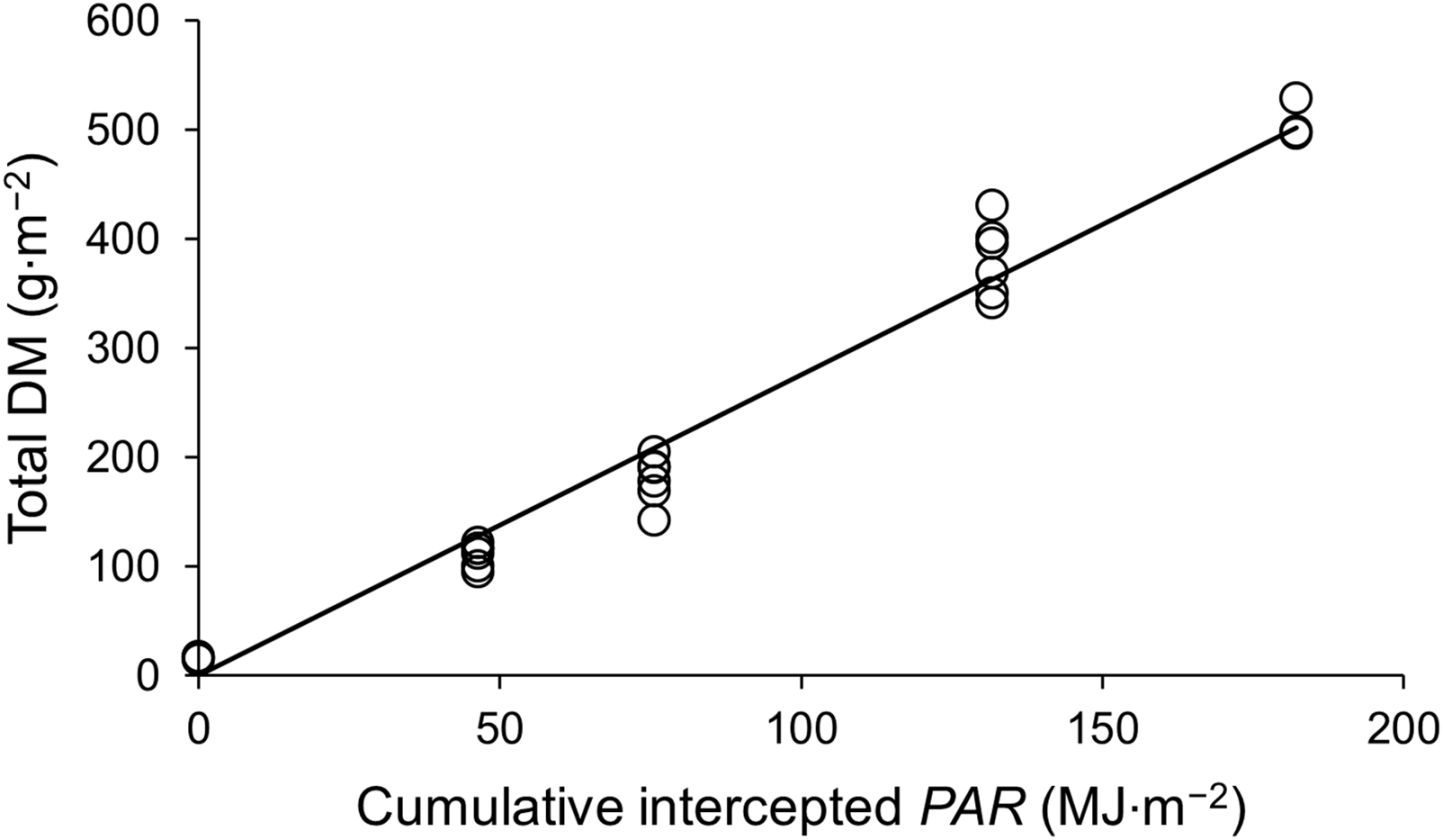
Total aboveground dry matter production as a function of cumulative intercepted *PAR* by strawberry plants.

**Table 1** shows the equations used in the model. DM production was calculated based on photosynthetically active radiation (*PAR*), the fraction of intercepted light, and light use efficiency (LUE; Higashide and Heuvelink, 2009). The fraction of intercepted light, or solar radiation through the canopy, was determined by the leaf area index (LAI) and light-extinction coefficient (*k*; Eq. 2). Notably, when using iLAI as the input variable, *k* is not required (Eq. 2’). LUE was determined from the linear regression of total DM production as a function of the interception of the integrated *PAR* on the sampling dates (**Figure 2**; Van der Ploeg et al., 2007).

**Table 1:** Model equations.

No.	Variable	Unit	Definition	Equation
(1)	*PAR_n_*	MJ·m^−2^	*PAR* at *n* DAT	$PAR_{n}=Sr_{n}\times 0.5$
(2)	*I_n_*	MJ·m^−2^	Intercepted light at *n* DAT	$I_{n}=PAR_{n}\times \left(1-exp^{-kLAI_{n}}\right)$
(2')	*I_n_*	MJ·m^−2^	Intercepted light at *n* DAT	$I_{n}=PAR_{n}\times iLAI$
(3)	Δ*DM_n_*	g·m^−2^	DM production at *n* DAT	$\text{Δ}DM_{n}=I_{n}\times \text{LUE}$
(4)	*TDM_n_*	g·m^−2^	Total DM at *n* DAT	$TDM_{n}=TDM_{\left(n-1\right)}+\text{Δ}DM_{n}$
(5)	*CT_n_*	°C	Cumulative temperature at *n* DAT from transplanting	$CT_{n}=\sum_{0}^{n}T_{n}$
(6)	*RLG_p_n_*		Relative growth of leaf number *p* at *n* DAT	$RLG_{p\_n}=1-1/\left[1+exp^{-\left\{\left({\text{CT}}_{n}-160p)-218.86\right)\right\}/-74.61}\right]$
(7)	Δ*RLG_p_n_*		Increment of leaf number *p* per day at *n* DAT	$\text{Δ}RLG_{p\_n}=RLG_{p\_n}-RLG_{p\_\left(n-1\right)}$
(8)	*TF_q_n_*	°C	Cumulative temperature from the first flower anthesis of *the qth* inflorescence	
(9)	*RFG_q_n_*		Relative growth of *the qth* inflorescence at *n* DAT	$RFG_{q\_n}=1/\left\{1+4615.91exp^{-\left(0.011\times {\text{TF}}_{q_{n}}\right)}\right\}$
(10)	Δ*RFG_q_n_*		Increment of *q* th fruit cluster per day at *n* DAT	$\text{Δ}RFG_{q\_n}=25\times \left\{RFG_{q\_n}-RFG_{q\_\left(n-1\right)}\right\}$
(11)	*GS_n_*	g·fruit clusters^−1^	Generative sink strength at *n* DAT	$GS_{n}=0.24\times \sum_{i=1}^{q}\text{Δ}RFG_{i\_n}$
(12)	*VS_n_*	g·tissue^−1^	Vegetative sink strength at *n* DAT	$VS_{n}=1.3\times \left(0.07\times \sum_{i=1}^{p}\text{Δ}RLG_{i\_n}\right)$
(13)	*AF_n_*	g·g^−1^	DM distribution to the fruit cluster at *n* DAT	$AF_{n}=GS_{n}/(VS_{n}+GS_{n})$
(14)	*DMF_q_n_*	g·fruit cluster^−1^	DM available for *the qth* fruit cluster at *n* DAT	$DMF_{q\_n}=\text{Δ}DM_{n}/\text{Pd}\times AF_{n}\times \left(\text{Δ}RFG_{q\_n}/\sum_{i=1}^{q}\text{Δ}RFG_{i\_n}\right)$
(15)	*DF_q_n_*	g·fruit cluster^−1^	DW of *the qth* fruit cluster at *n* DAT	$DF_{q\_n}=DMF_{q\_\left(n-1\right)}+DMF_{q\_n}$
(16)	*FF_q_n_*	gFW·fruit cluster^−1^	FW of *the qth* fruit cluster at *n* DAT	$FF_{q\_n}=DF_{q\_n}/\text{DMC}$
(17)	*F_n_*	gFW·plant^−1^	FW of fruit clusters at *n* DAT	$F_{n}=\sum_{i=1}^{q}FF_{q\_n}$
(18)	*Y_n_*	kgFW·m^−2^	Yield per unit at *n* DAT	$Y_{n}=\sum_{0}^{n}F_{n}\times \text{Pd}/1000$

The model calculated DM distribution to sink and source organs by determining the relative growth increments of leaf and fruit clusters (fruits from the same inflorescence). Relative leaf growth (RLG) was modeled based on cumulative temperature after leaf appearance, using the function of Chen et al. (2009). Leaf length at the end of elongation was rescaled to 1, and the relative leaf length was plotted to obtain the growth curve. Defining the leaf emergence interval as 160°C, the RLG of leaf number p was expressed in Eq. 6. Multiple inflorescences emerge irregularly from the crown, but we predefined that all inflorescences or fruit clusters grew similarly in response to temperature from the first flower anthesis. The cumulative yield at final harvest of each fruit cluster was rescaled to 1, and relative weights were plotted as a function of cumulative temperature post-anthesis to create Eq. 9. Individual growth increments of leaves (ΔRLG; Eq. 7) and fruit clusters (ΔRFG; Eq. 10) were derived from Eqs. 6 and 9, respectively. ΔRFG was adjusted based on the expected final harvested fruit weight of 250 g per fruit cluster, multiplied by the DW equivalent of 25 g.

DM distribution to the fruit cluster (AF) was calculated by dividing the generative sink strength (GS) by the sum of GS and vegetative sink strength (VS; Heuvelink, 1997). GS represents the daily growth of each fruit cluster multiplied by its sink strength (Eq. 11), defined as the ratio of remaining fruit DW to plant DW at maximum fruit retention, averaged from destructive measurements at 92 and 148 DAT. VS, including leaves, crown, and peduncles, was calculated by multiplying daily growth of each remaining leaf by its sink strength and then adjusting by the reciprocal of the ratio of leaf DW to vegetative parts DW (Eq. 12). Sink strength for leaves was the ratio of remaining leaf DW to plant DW, averaged over destructive measurements at 0, 55, 92, 148, and 182 DAT.

Daily available photoassimilates were distributed according to sink strength relative to total sink strength (de Koning, 1994; Heuvelink, 1996). The DW of each fruit cluster (DF) increased based on available DM for each fruit cluster (DMF), calculated from ΔDM per plant, AF, and ΔRFG relative to total ΔRFG (Eqs. 14 and 15). Since these calculations used DM, DMC was applied to convert DF to fresh weight (FF) of each fruit cluster (Eq. 16). Finally, the total fruit weight (F) was multiplied by plant density (Pd) to determine yield per unit area (Y; Eqs. 17 and 18). A yield simulation model incorporating all the equations was developed using Python.

### 2.3 Model validation

The model was validated for the typical forcing culture season in Japan, from September to April. We chose the growing season because most of strawberries in Japan are produced by forcing and semi-forcing culture (Yamasaki, 2013), which allows harvesting from winter to spring. Yields were validated over two growing seasons (2021–2022 and 2022–2023). To analyze the factors contributing to the differences between the simulated and observed values, total DM (TDM) and DM distribution to fruits were examined leading up to the yield output of the 2021–2022 season. As for the model inputs, we examined whether iLAI, which does not require plant-destructive measurements, could replace LAI. All simulations were conducted in the Jupyter notebook environment using Python. The model was validated using the following methods of data acquisition and analysis.

#### 2.3.1 General conditions in the greenhouse

‘Benihoppe’ plants were grown over two growing seasons: September 18, 2021 to April 25, 2022 (2021–2022) and September 26, 2022 to April 2, 2023 (2022–2023). Validation took place at the same location and with the same methods as described in Section 2.1. In brief, plants in 7.5-cm pots were maintained in a greenhouse and then transplanted into planters at a density of 7.05 plants·m^−^² on each planting date. The experiment followed a random block design with three replicates, and all plots were surrounded by strawberry plants at the same density.

#### 2.3.2 Data acquisition for model input variables

**Figure 3:**
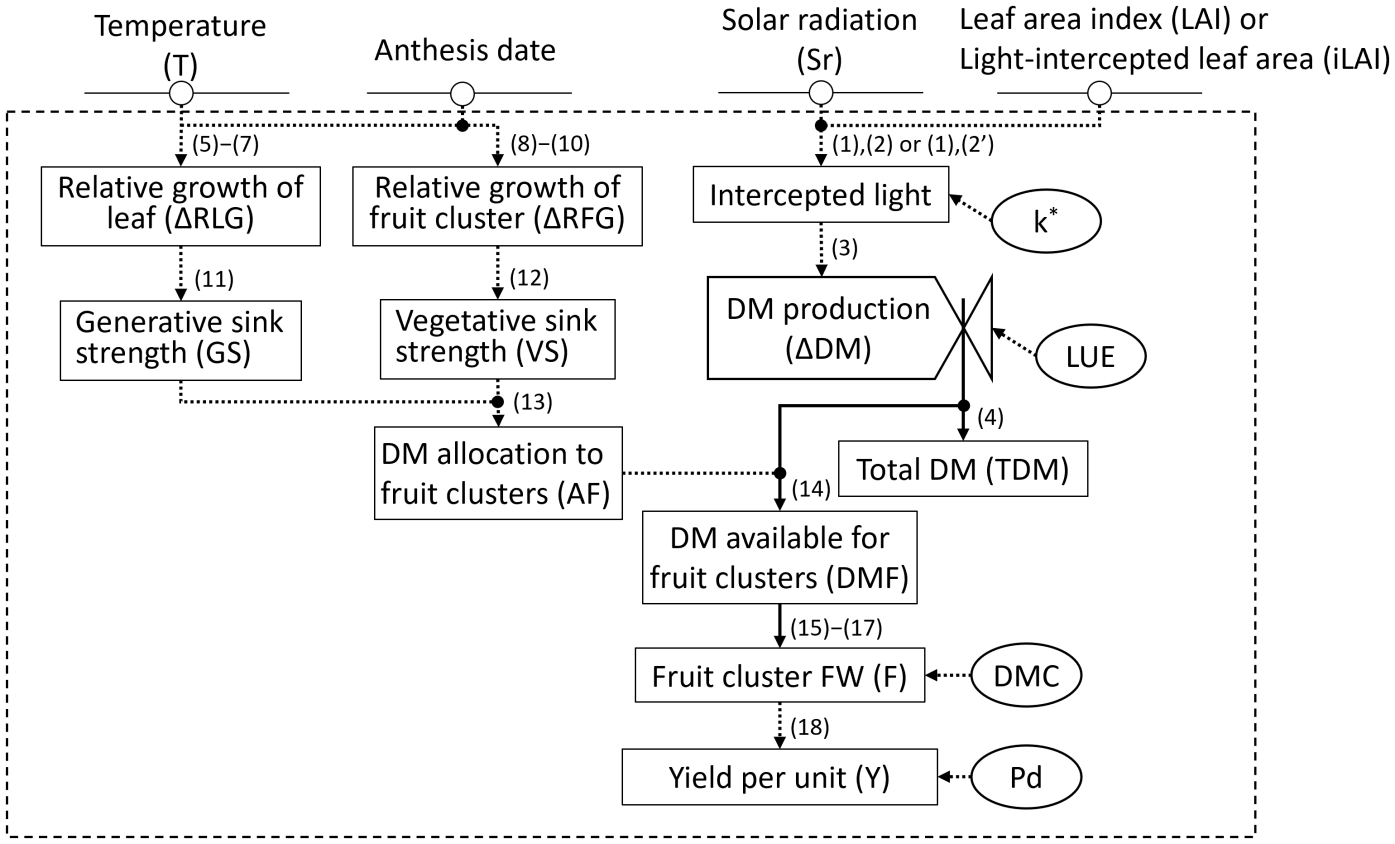
Forrester diagram for the greenhouse strawberry growth model: inputs, outputs, state variables, and parameters. State variables are shown by rectangles, rate variables by valves, input variables with a circle and horizontal line, and parameters with a circumference. A parameter with an asterisk indicates an optional parameter that is not necessary when calculated using Eq (2'). Material flows are represented by normal arrows, and information flows are represented by dashed arrows.

Model input variables included daily average temperature (°C) and solar radiation in the greenhouse (MJ·m^−^²), first flower anthesis date for each inflorescence, and LAI (m²·m^−^²) or iLAI (m²·m^−^²; **Figure 3**). The environmental data in the greenhouse were recorded by the Priva office. Three replicates of four plants per plot were used to evaluate flowering. The date of first flower anthesis for each inflorescence was recorded 2–3 times a week. To obtain the observed LAI, 2–4 plants were randomly sampled, and their leaf areas were measured destructively at the same growth stages as in Section 2.1. As an alternative to destructive LAI measurement, iLAI was measured weekly using a web camera (C922 Pro Stream, Logitech) positioned 1.2 m above the plants. For determining iLAI, images of the light-intercepted leaf area of a 14-plant canopy were linearized using ImageJ-Fiji image analysis software (National Institutes of Health). A set of observed LAIs or iLAIs was determined by linear interpolation of the measured values between each measurement date. Among the parameters used for model validation (**Table 2**), initial dry matter production (TDM_0_) was obtained by calculating the average of the above-ground DW of five transplants measured at the planting date.

**Table 2:** Model validation parameters.

Parameter	Symbol	Value (Reference)	
		2021–2022 growing season	2022–2023 growing season
Light-extinction coefficient	*k*	0.85 (Estimated)	
Light use efficiency	LUE	2.75 g·MJ^−1^ (Measured in 2019)	
Dry matter content of the fruit	DMC	0.10 g·g^−1^ (Measured in 2019)	
Initial dry matter production	TDM_0_	19.9 g·m^−2^	19.0 g·m^−2^
Plant density	Pd	7.05 plant·m^−2^	

#### 2.3.3 Data acquisition and analysis for model verification

Three replicates of four plants per plot were used to evaluate the yields. Fresh fruit weights were measured thrice weekly. To obtain the observed DW of fruits and TDM, five destructive measurements were conducted during the 2021–2022 season, as described in section 2.1. Site-specific DW of the aboveground parts of plants, whose LAI was measured as described in section 2.3.2, and the DW of pruned leaves were measured. The DM distribution to fruits over the interval between the dates of the five destructive measurements (AF’, g·g^−^¹) was calculated as follows:


AF'=(DFi+1−DFi)/{(TDMi+1−TDMi)/Pd} (1≤i≤5)

where DF*_i_* and TDM*_i_* are the total fruit DW and total DM on each date of the destructive measurements, respectively.

The model performance was evaluated using statistical indicators such as R², RMSE, and RRMSE. The RMSE and RRMSE were calculated as follows:


$$\text{RMSE}=\frac{\sqrt{\sum_{i=1}^{n}{\left(S_{i}-O_{i}\right)}^{2}}}{n}$$

$$\text{RRMSE}=\frac{\sqrt{\sum_{i=1}^{n}{\left(S_{i}-O_{i}\right)}^{2}/n}}{O_{mean}}$$

where *S_i_* and *O_i_* are the simulated and observed values, respectively, *O_mean_* is the observed mean, and *n* is the number of samples.

## 3 Results

### 3.1 Model overview

**Figure 3** provides an overview of the strawberry yield prediction model illustrated as a Forrester diagram. TDM is calculated based on intercepted light, which factors in solar radiation and leaf area (Higashide, 2022). For intercepted light, the light extinction coefficient (*k*) related to canopy solar radiation fraction is omitted when using iLAI rather than LAI. Sink strength, derived from individual leaf and fruit cluster growth, dictates dry matter (DM) distribution to fruit clusters (AF). Since fruit cluster weight is expressed as dry weight (DW) per plant, the yield per unit area (Y) is ultimately calculated using DM content (DMC) and plant density (Pd).

### 3.2 Analysis of the model performance

**Figure 4:**
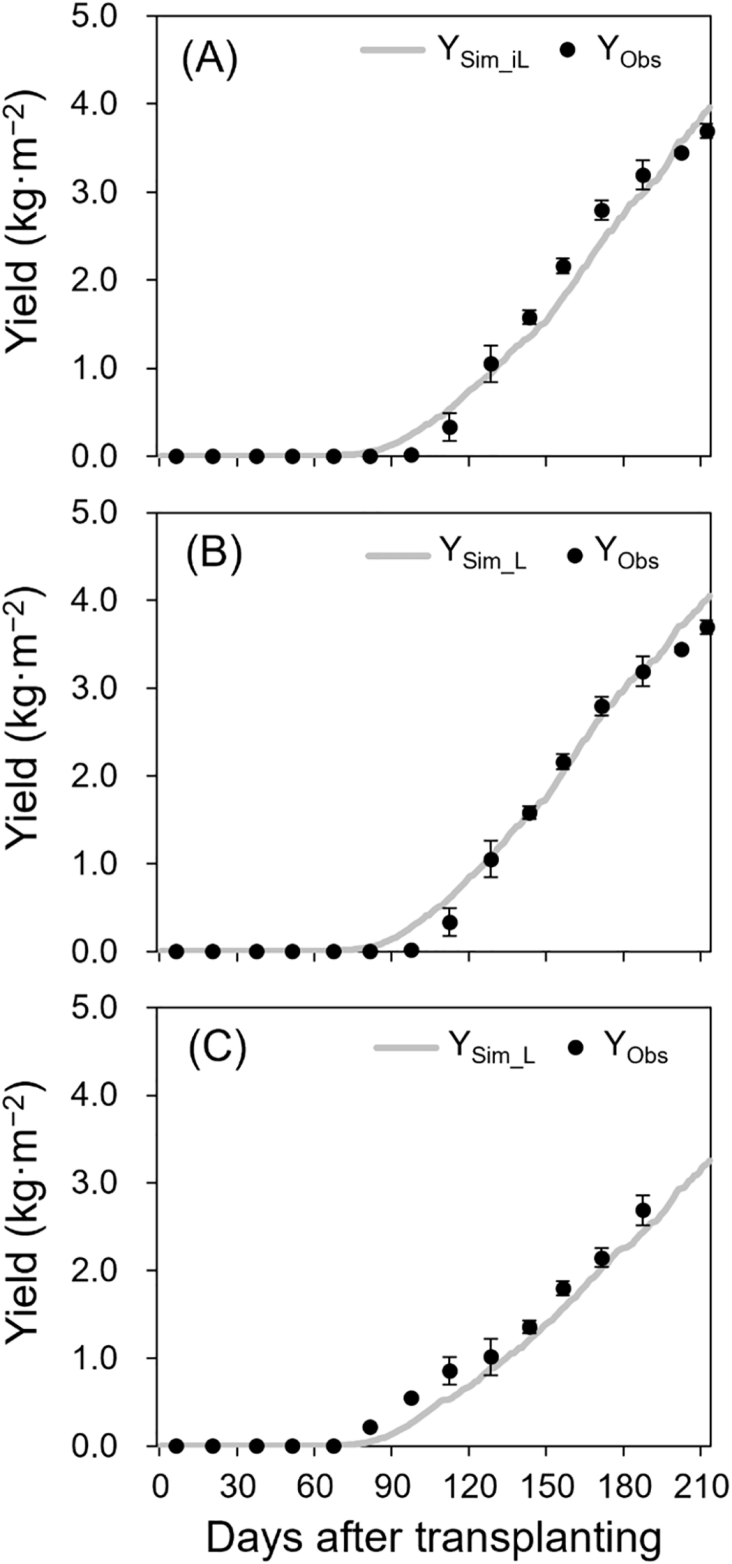
Simulated (lines) and observed (points ± SE) yields of the 2021–2022 growing season without destructive measurements **(A)**, 2021–2022 growing season with destructive measurements **(B)**, and 2022–2023 growing season with destructive measurements **(C)**. Y_Sim_iL_ in **(A)** and Y_Sim_L_ in **(B)** and **(C)** were simulated using iLAI and LAI, respectively. The points are the means of three replicates with four plants at biweekly intervals after transplanting.

In the 2021–2022 season, simulated yields using measured iLAI (Y_Sim_iL_, R² = 0.97, RRMSE = 0.14, RMSE = 0.23 kg·m^−^²; **Figure 4A**) and LAI (Y_Sim_L_, R² = 0.99, RRMSE = 0.11, RMSE = 0.18 kg·m^−^²; **Figure 4B**) showed strong alignment with observed yields (Y_Obs_). For the 2022–2023 season using measured LAI, the R², RRMSE, and RMSE values were 0.99, 0.15, and 0.20 kg·m^−^², respectively (**Figure 4C**).

**Figure 5:**
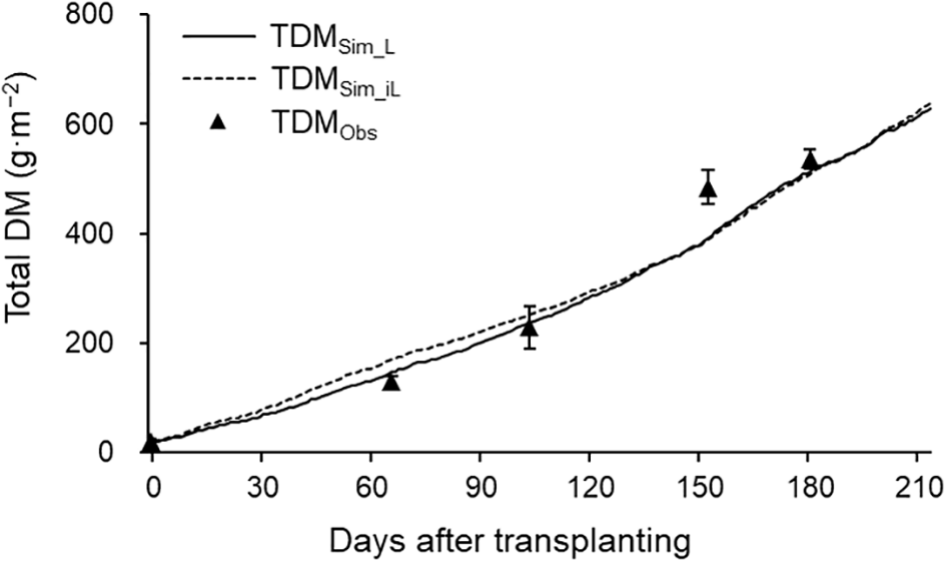
Simulated (lines) and observed (points ± SDs) total DM production in 2021–2022 growing season. TDM_Sim_L_ (solid line) and TDM_Sim_iL_ (dashed line) were simulated using LAI and iLAI, respectively. The points are the means of six plants at 0, 66, 104, 153, and 181 DAT.

**Figure 6:**
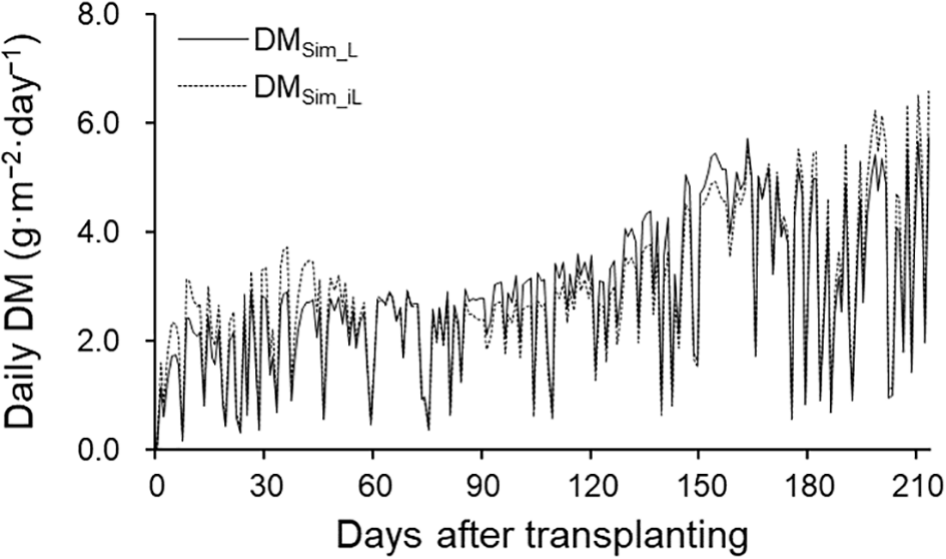
Fluctuations in simulated daily DM production using LAI (DM_Sim_L_; solid line) and iLAI (DM_Sim_iL_; dashed line) in the 2021–2022 growing season.

TDM and DM distribution analysis were performed using the 2021–2022 season data. Simulated TDM using LAI (TDM_Sim_L_) and iLAI (TDM_Sim_iL_) correlated well with observed TDM (TDM_Obs_), achieving R² values of 0.98 and 0.96, respectively (**Figure 5**). RRMSE and RMSE were 0.15 and 40.74 g·m^−^² for TDM_Sim_L_, and 0.17 and 46.93 g·m^−^² for TDM_Sim_iL_. Although final TDM_Sim_L_ and TDM_Sim_iL_ values were 628.17 g·m^−^² and 639.07 g·m^−^², respectively, DM_Sim_iL_ showed lower daily DM production than DM_Sim_L_ from 70 to 165 DAT, during flowering and harvesting (**Figure 6**), with a cumulative 30.59 g difference.

**Figure 7:**
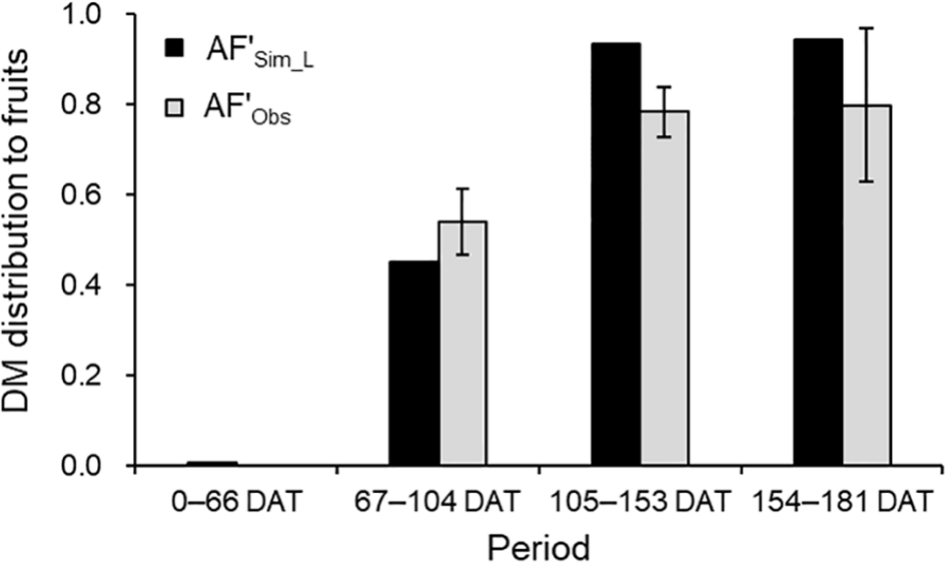
Comparison of simulated (AF'_Sim_L_) and observed (AF'_Obs_) DM distributions to fruits during each period between the destructive measurements in 2021–2022 growing season. The observed values are the mean ± SD (*n* = 6).

Analysis using LAI-based simulations for TDM showed satisfactory accuracy for AF’ and monthly yield. Observed AF’ (AF’_Obs_) was 0.54 from flowering to the start of the 1st inflorescence harvest (67–104 DAT) and 0.80 during the 2nd and 3rd inflorescence harvests (105–153 DAT and 154–181 DAT; **Figure 7**). Simulated AF’ (AF’_Sim_L_) reflected similar trends, closely aligning with AF’_Obs_ ± SD at 154–181 DAT.

**Figure 8:**
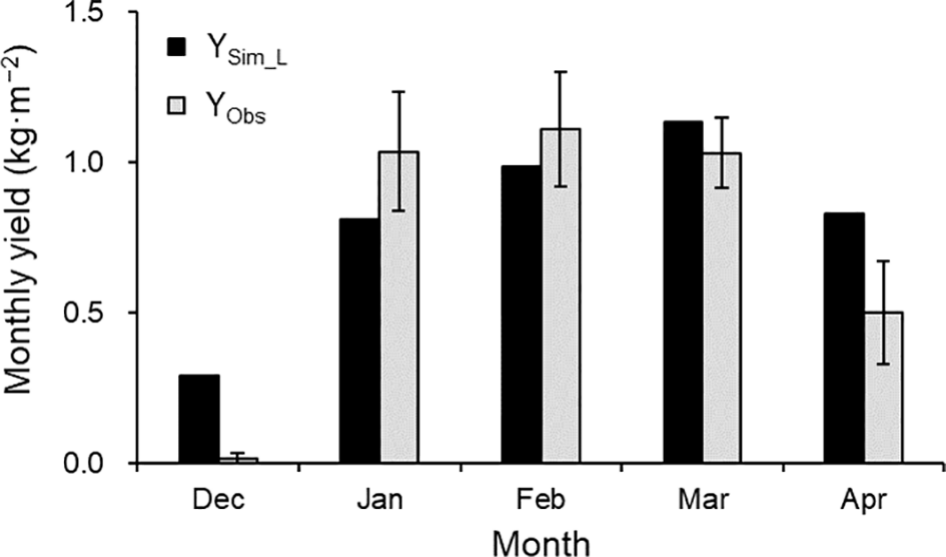
Comparison of simulated (Y_Sim_L_) and observed (Y_Obs_) monthly yields in the 2021–2022 growing season. The observed values are the mean ± SE (*n* = 3).

Given the satisfactory TDM and AF’ simulations, the simulated monthly yield generally matched observed values from January to March (**Figure 8**). However, simulated yields were overestimated in December and April, at the start and end of harvest.

## 4 Discussion

### 4.1 Model performance

This study showed good agreement between simulated and observed strawberry yields across two distinct growing seasons, highlighted by low RRMSE and RMSE values. For the 2021–2022 growth dataset, the simulated TDM and DM distribution closely matched observed values, supporting accurate yield simulations except the beginning and end of the growing season.

DM assimilation through photosynthesis and its distribution are critical for crop productivity (Forney and Breen, 1985). In this study, the RRMSE of TDM was reduced to 0.15 by incorporating measured LAI values, improving on the 0.26 reported in Hopf et al. (2022a). Even using iLAI without destructive measurements yielded a respectable RRMSE of 0.17. The web camera images of upper leaves, not accounting for canopy structure or height, may have caused some underestimation of photosensitive leaf area, especially in ‘Benihoppe’ plants with upright petioles that facilitate sunlight penetration (Mochizuki et al., 2013; Takahashi et al., 2020). This underestimation could reduce daily DM production, as shown in **Figure 6**.

DM distribution between vegetative and generative growth is guided by the vegetative-to-fruit unit ratio (de Koning, 1994). In strawberries, such as ‘Benihoppe,’ leaves between inflorescences range from 3 to 4, with varying optimal numbers across cultivars (Takeuchi and Sasaki, 2008). Here, vegetative units included leaves, crowns, and peduncles, while fruit clusters on each inflorescence served as generative units. Temperature-driven leaf emergence intervals and individual growth curves were the growth calculations for the leaves, and fruit cluster growth calculation was the growth curve starting from a manually recorded flowering date. During the harvest period, the DM distribution to fruits was simulated at 80%, aligning with the 80% distribution to fruits and peduncles observed by Mochizuki et al. (2013) in ‘Benihoppe’ (**Figure 7**). This reflects a consistent DM distribution trend, reinforcing the model’s reliability in predicting DM distribution dynamics in strawberry cultivation.

### 4.2 Future work

The versatility of the model can be enhanced by: 1) incorporating optimal temperature thresholds, 2) implementing a dynamic LUE parameter, and 3) developing cultivar-specific coefficients for different cultivars.

In Japan, the forcing culture typically continues harvesting until May, which extends beyond this study’s validation period. In the 2021–2022 season, the daily average temperature was within the range of 9.8°C–25.5°C, but the instantaneous values exceeded 30°C at times, beyond the optimal temperature range (Hellman and Travis, 1988; Galletta and Himelrick, 1990) at the beginning and end of the growing seasons. Hopf et al. (2022a) incorporated the difference between actual and cardinal (optimal) temperatures into their fruit growth calculations, noting that cardinal temperatures contributed to reducing excessive early fruit production in the yield simulation. Considering cardinal temperatures in our model may reduce the overestimation of yield at the beginning and end of the growing season (**Figure 8**). Moreover, it may enable longer simulation periods.

During seasons with low solar radiation, such as winter, strawberry plants are grown in CO_2_-enriched greenhouses to significantly enhance photosynthesis and yield (Miyoshi et al., 2017; Tagawa et al., 2022). In our model, LUE was used as a constant, assuming a greenhouse CO_2_ concentration of 400 ppm. By contrast, LUE was dynamically adjusted in response to varying CO_2_ levels in the tomato model (Higashide and Heuvelink, 2009). In wheat, the plant developmental stage and air temperature affected LUE throughout the growing season (Song et al., 2022). These studies provide enough evidence for the necessity of dynamically adjusting the LUE.

Multiple studies have compared the plant growth and fruit production of commercially used Japanese cultivars grown in various environments (Mochizuki et al., 2013; Hidaka et al., 2015; Nakayama and Nakazawa, 2023). Considering these reports, the leaf and fruit growth parameters presented in this study may vary among cultivars. Therefore, the generalizability of the model will be enhanced by evaluating the determined parameters in ‘Benihoppe’ across different cultivars or by recalibrating the parameters as necessary for each cultivar.

While the present model can be improved, it is applicable to strawberry production in greenhouses in hydroponic facilities, given that the input data for the model are available. Because strawberry yields fluctuate dramatically over short periods such as a week, yield simulations would help growers determine resource needs and plant management strategies (MacKenzie and Chandler, 2009; Chen et al., 2019). In conclusion, the model provides growers with a valuable tool for yield forecasting tailored to the specific conditions within their greenhouse, facilitating effective resource planning from harvest through marketing.

## Data Availability

The original contributions presented in the study are included in the article/supplementary material. Further inquiries can be directed to the corresponding author/s.
